# *T*_1_-Weight Magnetic Resonance Imaging Performances of Iron Oxide Nanoparticles Modified with a Natural Protein Macromolecule and an Artificial Macromolecule

**DOI:** 10.3390/nano9020170

**Published:** 2019-01-30

**Authors:** Cheng Tao, Qiang Zheng, Lu An, Meie He, Jiaomin Lin, Qiwei Tian, Shiping Yang

**Affiliations:** The Key Laboratory of Resource Chemistry of the Ministry of Education, the Shanghai Key Laboratory of Rare Earth Functional Materials, and the Shanghai Municipal Education Committee Key Laboratory of Molecular Imaging Probes and Sensors, Shanghai Normal University, Shanghai 200234, China; 1000441586@smail.shnu.edu.cn (C.T.); diwuxiaoo@163.com (Q.Z.); anlu1987@shnu.ed.cn (L.A.); 1000441337@smail.shnu.edu.cn (M.H.); qiweitian@shnu.edu.cn (Q.T.)

**Keywords:** magnetic resonance imaging, *T*_1_-weight contrasts, magnetic nanoparticles, iron oxide, macromolecule ligands

## Abstract

To optimize the iron oxide nanoparticles as *T*_1_-weight contrast for in vivo magnetic resonance imaging (MRI), numbers of macromolecule ligands have been explored with considerable effort. However, reports refer to the comparison of the *T*_1_-weight contrast performances of iron oxide nanoparticles modified with natural and artificial macromolecule ligands are still limited. In this work, we used a typical natural protein macromolecule (bovine serum albumin, BSA) and an artificial macromolecule (poly(acrylic acid)-poly(methacrylic acid), PMAA-PTTM) as surface ligands to fabricate Fe_3_O_4_-BSA and Fe_3_O_4_-PMAA-PTTM nanoparticles with similar size and magnetization by the coprecipitation method and compared their MRI performances. In vitro and in vivo experiments revealed that Fe_3_O_4_-BSA with lower cytotoxicity exhibited higher *r*_2_/*r*_1_ ratio in solution and darkening contrast enhancement for liver and kidney sites of mice under *T*_1_-weight imaging, while Fe_3_O_4_-PMAA-PTTM displayed much lower *r*_2_/*r*_1_ ratio in solution and brighter contrast enhancement for liver and kidney sites. These remarkably different MRI behaviors demonstrated that the surface ligands play an important role for optimizing the MRI performance of Fe_3_O_4_ nanoparticles. We expect these results may facilitate the design of macromolecule ligands for developing an iron oxide–based *T*_1_-weight contrast agent.

## 1. Introduction

Magnetic resonance imaging (MRI) has become one of the most powerful imaging techniques because of its capacity to non-invasively render images with high temporal and spatial resolution [1,2]. To obtain high-quality imaging of diseases, contrast agents are often required during MRI scans. Generally, contrast agents are divided into *T*_1_-weighted contrast, which is able to facilitate the spin-lattice relaxation of protons to generate positive (or bright) contrast enhancement, and *T*_2_-weighted contrast, which is able to cause protons in their vicinity to undergo spin–spin relaxation to produce negative (or dark) contrast enhancement [1]. Due to these intrinsic shortcomings (such as false signal from the calcification and metal deposition areas) of *T*_2_-weighted imaging, *T*_1_ contrast agents are general more preferred clinical diagnostic agents [3]. Currently, the *T*_1_ MRI contrast agents is mainly dominated by the paramagnetic Gd-chelates [4] (including Magnevist (Gd-DTPA), Dotarem (Gd-DTOA), and Eovist (Gd-EOB-DTPA)) and some Mn-based compounds [5,6] and nanoparticles [7]. However, because of the potential risk of these Gd- and Mn- ions in vivo (e.g., nephrogenic systemic fibrosis caused by the release of the Gd ions from the chelate ligands [8]), great efforts have been devoted to develop alternative *T*_1_ contrast agents. 

Magnetic iron oxide nanoparticles, which are usually used as *T*_2_ contrast agents [9,10,11,12,13,14], have recently been proved to be potential *T*_1_ contrast agents when their sizes are down to small diameter (usually <5 nm) [15,16,17,18,19,20,21,22]. These small iron oxide nanoparticles can display excellent *T*_1_ contrast performance owning to their large surface area with five unpaired electrons of iron ions. Besides, because iron ions are naturally found in the human body and serve as an important physiological element for hemoglobin, iron oxide is considered more biocompatible as compared with Gd- and Mn-based compounds and nanoparticles [18,23]. However, the utilization of iron oxide nanoparticles as *T*_1_ contrast agents still remains a significant challenge because they tend to form aggregations under physiological conditions and thus result in intensive *T*_2_ contrast enhancement and the disappearance of *T*_1_ contrast enhancement [24]. To overcome this challenge, great efforts have recently been devoted to explore hydrophilic macromolecule to modify the surface of iron oxide nanoparticles through different approaches [25,26,27]. Tailoring the surface of nanoparticles with hydrophilic ligands can avoid the aggregation in vivo and also helps to improve their stability, biocompatibility, and in vivo circulation time [28,29]. Therefore, the development of surface ligands is very important for iron oxide-based contrast agent.

With considerable and substantial efforts in the past few years, a number of macromolecule ligands have been explored, and many of them were shown to be promising ligands for optimizing the *T*_1_-weight contrast performance of iron oxide nanoparticles [17,18,26,30,31,32,33,34]. Particularly, bovine serum albumin (BSA) [31,35] and poly(acrylic acid)-poly(methacrylic acid) (PMAA-PTTM) [32,36] represent typical natural protein macromolecules and artificial macromolecules with abundant coordination groups that can interact with metal ions, which have been widely used as surface ligands for modifying nanoparticles for biological application. For example, Li et al. reported a BSA-modified iron oxide nanoparticle with good biocompatibility for in vivo *T*_1_ and *T*_2_-weighted imaging [31]. Lu et al. reported a PMAA-PTTM modified iron oxide nanoparticle showed high water-soluble and good performance of *T*_1_ and *T*_2_-weighted imaging [32]. Nevertheless, reposts refer to the comparison of *T*_1_-weight contrast performances of iron oxide nanoparticles functionalized with these two macromolecules are still limited. In this work, two kinds of iron oxide nanoparticles with similar size and magnetization and surface ligands modified with BSA and PMAA-PTTM, respectively, were fabricated through coprecipitation method, and their cytotoxicity and *T*_1_-weight contrast performances were compared through in vitro and in vivo experiments.

## 2. Materials and Methods

### 2.1. Materials

FeCl_3_·6H_2_O, Fe(SO_4_)_2_·7H_2_O, ammonia solution (25%) were obtained from Sigma Aldrich (Shanghai, China). Bovine serum albumin (BSA) was purchased from Amresco (Solon, OH, USA). All reagents were used without further purification. Poly(acrylic acid)-poly(methacrylic acid) (PMAA-PTTM) was prepared according to the reference [36]. Mouse selection and methods of operations were performed in strictly according to the requirements of the Animal Ethics Committee of the Shanghai Normal University and the Institutional Animal Care and Use Committee.

### 2.2. Synthesis of Fe_3_O_4_-BSA and Fe_3_O_4_-PMAA-PTTM Nanoparticles

In a 250 mL three-necked flask, the ligand BSA (138 mg) was added into 50 mL of deionized water and then stirred under nitrogen atmosphere for one hour to remove the oxygen. Then, a solution (2 mL) containing FeCl_3_·6H_2_O (0.082 mmol, 22.3 mg) and Fe(SO_4_)_2_·7H_2_O (0.25 mmol, 70 mg) was injected into the above solution and heated to 90 °C for 5 min, followed by the injection of 5 mL of concentrated ammonia solution under stirred. The mixed solution becomes black immediately, and the reaction was kept at 90 °C for 2 h before cooled down to room temperature. The Fe_3_O_4_-BSA nanoparticles were obtained through ultrafiltration centrifugation with a 10-k ultra-filtration centrifuge tube for 7–8 times to remove impurities. The finally obtained Fe_3_O_4_-BSA nanoparticles were dispersed in deionized water and stored at 5 °C for further experiments.

The Fe_3_O_4_-PMAA-PTTM nanoparticles were prepared similar with that of Fe_3_O_4_-BSA, except the ligand of BSA was replaced by PMAA-PTTM. The final black solution was also ultrafiltration centrifuged with a 10-k ultra-filtration centrifuge tube for 7–8 times to remove the impurities, and the obtained Fe_3_O_4_-PMAA-PTTM nanoparticles were also dispersed in deionized water and stored at 5 °C for further experiments. 

### 2.3. Characterization

Powder X-ray diffraction (PXRD) data with scan range of 10–80° were collected on a Bruker X-ray powder diffractometer (D8 ADVANCE, Cu Kα, Brucker, Karlsruhe, Germany). Fourier-transform IR (FT-IR) spectra with recording wavenumber of 400–4000 cm^−1^ and potassium bromide as pressed pellets were recorded on a Nicolet Avatar 370 FT-IR spectrophotometer (Thermo Electron Corporation, Atkinson, NH, USA). Hysteresis loops were obtained from a superconducting quantum interference device (Lake Shore Cryotronics, Inc., Westerville, OH, USA). Transmission electron microscopy images for the nanoparticles were carried out via JEOL JEM-2010 microscopy (JEOL, Tokyo, Japan). The concentrations of iron-ion were determined through high-dispersion inductively coupled plasma atomic emission spectroscopy (ICP, Prodigy, Teledyne Leeman Labs Inc., Hudson, NY, USA). The longitudinal relaxation times (*T*_1_) and transverse relaxation times (*T*_2_) of Fe_3_O_4_-BSA and Fe_3_O_4_-PMAA-PTTM were measured with different iron ion concentrations on a 0.5 T magnetic resonance scanner (NMI20, Niumag, Shanghai, China) with parameters of SF, 18 MHz; TW, 8000 ms; SW, 100 kHz; RG, 20 db; DRG1, 3. The longitudinal relaxation rate (*r*_1_) and transverse relaxation rate (*r*_2_) were obtained by a linear fitting of the iron ion concentration and 1/*T*_1_ and 1/*T*_2_. *T*_1_-weighted phantom images of Fe_3_O_4_-BSA and Fe_3_O_4_-PMAA-PTTM with different concentrations were carried out on the same magnetic resonance scanner. The parameters for *T*_1_-weighted phantom images were following: repetition time (TR), 300 ms and echo time (TE), 0.04 ms.

### 2.4. In Vitro Cytotoxicity Assay

Mouse breast tumor 4T1 cells were used to assay the cytotoxicity of Fe_3_O_4_-BSA and Fe_3_O_4_-PMAA-PTTM nanoparticles. For this study, 4T1 cells were provided from the Shanghai Institutes for Biological Sciences and cultured in Dulbecco’s modified eagle medium at 37 °C with 5% CO_2_ and 100% humidity for 24 h. The cytotoxicity was evaluated with MTT assays. Typically, 4T1 cells (5 × 10^4^ cells/well) were first plated in 96-well plate for 24 h and then treated with different concentrations of Fe_3_O_4_-BSA or Fe_3_O_4_-PMAA-PTTM nanoparticles (0, 12.5, 25, 50, and 100 μg/mL) in DMEM for 12 or 24 h at 37 °C with 5% CO_2_. Thiazolyl blue tetrazolium bromide (20 μL, 5 mg/mL) was added to the well and further incubated for four hours. The supernatant of the plate was removed, and the remaining purple formazan crystals were lysed with 150 μL of dimethyl sulfoxide. The absorption of the formazan was determined through a microplate reader (Multiskan MK3, Thermo Fisher Scientific, Waltham, MA, USA) with a microplate reader (Thermo Fisher Scientific, Waltham, MA, USA) as background.

### 2.5. In Vivo Magnetic Resonance Imaging

In vivo *T*_1_-weighted images of mice were performed on a 0.5 T MRI scanner (Siemens Medical systems, Erlangen, Germany). During imaging, the mice with weigh of about 20 g were anesthetized by intravenous injection with 8% chloral hydrate. The mice were first imaged in different transversal slices to show the heart, liver, and kidney sites and acquire the images as control groups. The mice were then given an intravenous injection of Fe_3_O_4_-BSA with a dose of 10 mg [Fe]/Kg [mouse]. After that, time-scale images with time points of 0, 40, 90, and 180 min were obtained with the same transversal slices. The *T*_1_-weighted images of mice that received an intravenous injection of Fe_3_O_4_-PMAA-PTTM were carried out with the same procedures and injected dose, except the imaging time points were 20, 60, 120, 180, and 240 min. The imaging parameters were the following: (1) field of view, 80 × 80 mm, (2) repetition time (TR), 500 ms, (3) matrix size, 256 × 192 mm (4) echo time (TE), 18 ms, and (5) slice thickness, 3 mm. 

## 3. Results and Discussion

### 3.1. Synthesis and Characterizations

**Figure 1:**
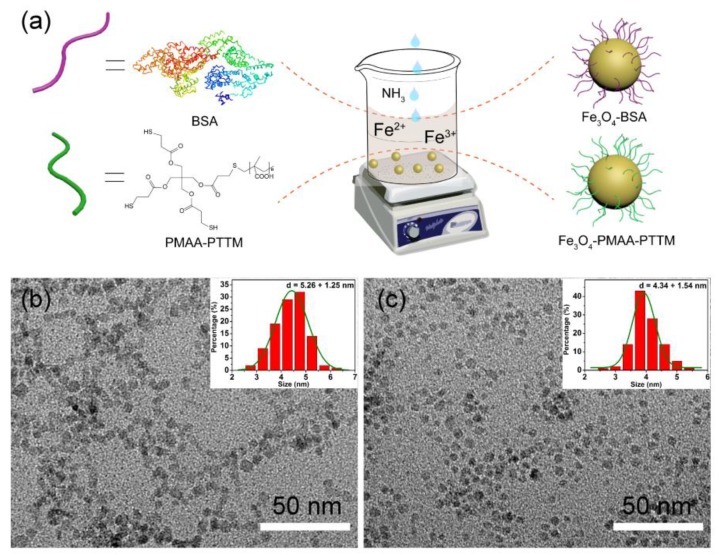
(**a**) Schematic illustration of the fabrication process of Fe_3_O_4_-BSA and Fe_3_O_4_-PMAA-PTTM nanoparticles; Transmission electron microscopy (TEM) images of (**b**) Fe_3_O_4_-BSA and (**c**) Fe_3_O_4_-PMAA-PTTM nanoparticles.

**Figure 2:**
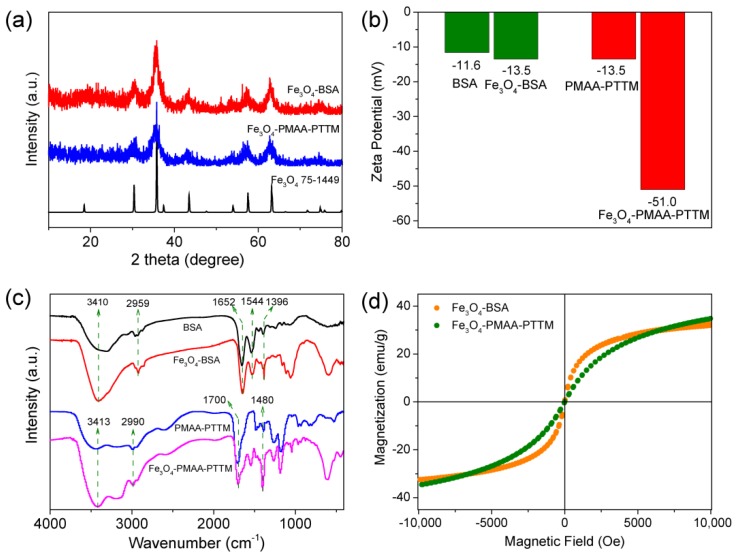
(**a**) Powder X-ray diffraction (PXRD) of Fe_3_O_4_-BSA (red line), Fe_3_O_4_-PMAA-PTTM (blue line) and a Fe_3_O_4_ powder standard (JCPDS Card No. 75-1449, black line); (**b**) Zeta potential of BSA, Fe_3_O_4_-BSA, PMAA-PTTM and Fe_3_O_4_-PMAA-PTTM in H_2_O; (**c**) Fourier-transform infrared spectroscopy of bovine serum albumin (BSA, black line), Fe3O4-BSA (red line), PMAA-PTTM (blue line) and Fe3O4-PMAA-PTTM (purple line); (**d**) Field-dependent magnetization curves for Fe_3_O_4_-BSA (yellow dot) and Fe_3_O_4_-PMAA-PTTM (green dot) nanoparticles at 298 K.

Coprecipitation is a convenient approach for fabrication of Fe_3_O_4_ nanoparticles because it can be carried out in aqueous solution and did not required further surfactant change of the as-synthesized nanoparticles to improve their water-solubility. The Fe_3_O_4_-BSA and Fe_3_O_4_-PMAA-PTTM nanoparticles were prepared through this approach with ferric and ferrous salt in the presence of ammonia solution (Figure 1a). Transmission electron microscopy (TEM) images show that the average diameters of Fe_3_O_4_-BSA and Fe_3_O_4_-PMAA-PTTM nanoparticles are 5.26 ± 1.25 and 4.34 ± 1.54 nm respectively, showing very similar size for both samples (Figure 1b,c). The narrow size distribution of both samples indicates that the BSA and PMAA-PTTM macromolecules are good templates and stabilizers for controlling the formation and growth of the Fe_3_O_4_ nanoparticles. The crystalline structures of the as-synthesized samples were determined by powder X-ray diffraction (PXRD). As shown in Figure 2a, the diffraction peaks of both samples are found to be well matched with that of the theoretical patterns of Fe_3_O_4_ (JCPDS No.75-1449), and no other peaks were detected, confirming the successful preparation of the pure phase Fe_3_O_4_. The obvious diffraction peaks of both samples indicate that the Fe_3_O_4_ nanoparticles prepared through such one-step coprecipitation approach have high crystallinity, which is important for magnetic nanoparticles. The zeta potential of Fe_3_O_4_-BSA and Fe_3_O_4_-PMAA-PTTM were determined to be −13.5 and −51.0 mV, respectively, which is consistent with the negative potential of BSA and PMAA-PTTM (Figure 2b).

The surface functionalization of the Fe_3_O_4_ nanoparticles with BSA and PMAA-PTTM ligands were confirmed by Fourier transform infrared (FT-IR) spectrum. As shown in Figure 2c, the BSA displayed characteristic wide absorption peck around 3000–3600 cm^−1^ (O–H and N–H stretching vibrations), and strong absorption peaks at 2959 cm^−1^ (–CH_2_– symmetric vibrations), 1652 cm^−1^ (C=O stretching vibrations, amide I), 1544 cm^−1^ (N–H stretching vibrations, amide II), and 1396 cm^−1^ (side chain COO^-^) [29]. All of these absorption pecks can also be observed in the FT-IR spectrum of Fe_3_O_4_-BSA, indicating that the Fe_3_O_4_ nanoparticles were surface decorated with BSA. For the PMAA-PTTM, characteristic absorption pecks were observed around 3000–3600 cm^−1^ (O–H stretching vibrations), 2990 cm^−1^ (–CH_2_– symmetric vibrations), 1700 cm^−1^ (C=O stretching vibrations, amide I), and 1480 cm^−1^ (C–O stretching vibrations of COO^-^) [30]. These absorption pecks can be also observed in that of the Fe_3_O_4_-PMAA-PTTM, demonstrating the presence of PMAA-PTTM macromolecules in the nanoparticles. Besides, the absorption at the 1480 cm^−1^ for the asymmetric C–O stretching vibrations of carboxyl group was lightly shifted to 1550, indicating that the carboxyl groups were coordinated with the Fe ions [22].

Superparamagetism is an important character for Fe_3_O_4_ nanoparticles to be used as MRI contrast agents. The magnetization curve of Fe_3_O_4_-BSA and Fe_3_O_4_-PMAA-PTTM were measured at room temperature using a superconducting quantum interference device with magnetic field up to 1.0 T. As shown in Figure 2d, the coercivity and remanence in hysteresis loops for both Fe_3_O_4_-BSA and Fe_3_O_4_-PMAA-PTTM are negligible, indicating they exhibit paramagnetic behavior at room temperature. The saturated magnetization values were determined to be 31.8 and 34.5 emu/g for Fe_3_O_4_-BSA and Fe_3_O_4_-PMAA-PTTM, respectively, giving very similar magnetization values for both materials. The similar intrinsic paramagnetic properties of Fe_3_O_4_-BSA and Fe_3_O_4_-PMAA-PTTM may enable more convenience for the comparison of their MRI performance. 

### 3.2. In Vitro Magnetic Resonance Imaging

**Figure 3:**
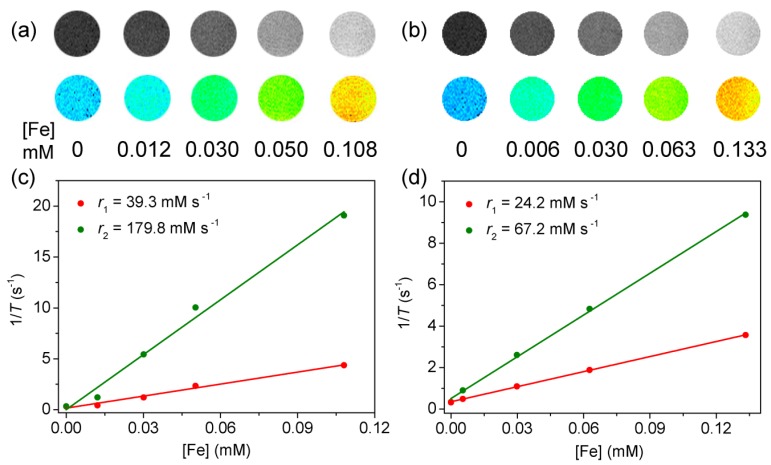
*T*_1_-weight phantom images of (**a**) Fe_3_O_4_-BSA and (**b**) Fe_3_O_4_-PMAA-PTTM nanoparticles with various Fe concentrations. Plot of 1/*T* over Fe concentration of (**c**) Fe_3_O_4_-BSA and (**d**) Fe_3_O_4_-PMAA-PTTM nanoparticles. The slopes indicate the relaxivity of *r*_1_ (red line) and *r*_2_ (green line).

To evaluate the MRI performance, the contrast enhancement properties and relaxation times (T) of Fe_3_O_4_-BSA and Fe_3_O_4_-PMAA-PTTM in aqueous solution with different concentrations were determined on a 0.5 T MRI scanner. As showed in Figure 3a–b, for the *T*_1_ phantom images, both Fe_3_O_4_-BSA and Fe_3_O_4_-PMAA-PTTM displayed brightening contrast enhancement with the increasing concentration of Fe, indicating that they were *T*_1_ contrast agent at these concentrationa. The longitudinal (*r*_1_) and transverse (*r*_2_) molar relaxivities were calculated according to the equation of *r* = ∆(1/T)/∆[Fe]. The *r*_1_ and *r*_2_ relaxivities of Fe_3_O_4_-BSA and Fe_3_O_4_-PMAA-PTTM were 39.3 and 179.8 mM^−1^·s^−1^ and 24.2 and 67.2 mM^−1^·s^−1^, respectively, corresponding to *r*_2_/*r*_1_ ratios of 4.58 and 2.78, respectively. The low *r*_2_/*r*_1_ ratios (below 5.0) of Fe_3_O_4_-BSA and Fe_3_O_4_-PMAA-PTTM confirmed that they are adequate for *T*_1_-weighted contrast agents. Compared with Fe_3_O_4_-BSA, Fe_3_O_4_-PMAA-PTTM possessed a lower *r*_2_/*r*_1_ ratio, indicating that it is a better *T*_1_ contrast agent [16]. Considering that both Fe_3_O_4_-BSA and Fe_3_O_4_-PMAA-PTTM possessed similar particle-sizes and saturated magnetization values, the different relaxivities should be mainly attributed to the effects of the surface decorated ligands. In this context, BSA is a natural protein macromolecule, which not only possessed hydrophilic groups such as carboxyl groups that can coordinated with Fe ions, but also contained abundant hydrophobic side groups that may restrict the diffusion and exchange of water protons (with the coordinated water molecules) surrounding the nanoparticles, and even induce the aggregation of the nanoparticles in the solution, consequently leading to large *r*_2_ relaxivity and *r*_2_/*r*_1_ ratio. In contrast, PMAA-PTTM is a hydrophilic macromolecule, which may be benefit for the surface diffusion and exchange of water protons, and also effectively prevent the aggregation of the nanoparticles, thus result in lower *r*_2_ relaxivity and *r*_2_/*r*_1_ ratio.

### 3.3. In Vitro Cytotoxicity

**Figure 4:**
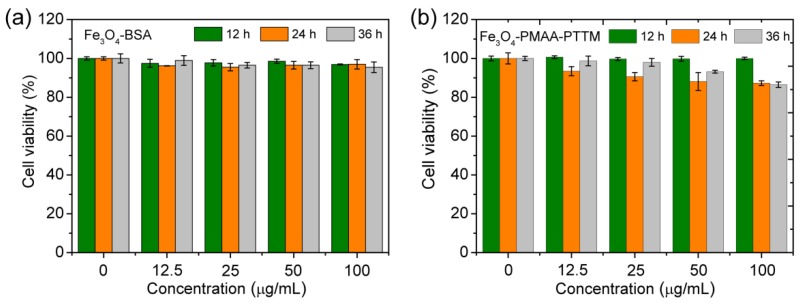
Cell viability of 4T1 cell line after incubation with different concentrations of (**a**) Fe_3_O_4_-BSA and (**b**) Fe_3_O_4_-PMAA-PTTM for 12, 24 and 36 h.

Before in vivo tests, the cytotoxicity of Fe_3_O_4_-BSA and Fe_3_O_4_-PMAA-PTTM were assessed by using a standard methyl thiazolyltetrazolium (MTT) assay with 4T1 (a mouse breast cancer cell line) cell lines. The cell viability after incubation with different concentration of nanoparticles (0–100 μg·mL^−1^ based on Fe) for 12, 24 and 36 h are showed in Figure 4a. For Fe_3_O_4_-BSA, the cell viability was almost not affected after incubation for 12 h with the Fe concentration up to 100 µg·mL^−1^. When the incubation time was prolonged to 36 h (with the same concentration of nanoparticles), the cell viability still remained above 95%. The cell that incubated with Fe_3_O_4_-PMAA-PTTM also has almost not affected after incubation for 12 h, while when the incubation time were extended to 36, the cell viability slightly dropped to 86% when the concentration of Fe was up to 100 µg·mL^−1^. These results indicate that both Fe_3_O_4_-BSA and Fe_3_O_4_-PMAA-PTTM (less than 100 µg·mL^−1^) have low cytotoxicity, but Fe_3_O_4_-BSA is more biocompatible as compared with Fe_3_O_4_-PMAA-PTTM, which is reasonable since BSA is a natural protein macromolecule.

### 3.4. In Vivo Magnetic Resonance Imaging

**Figure 5:**
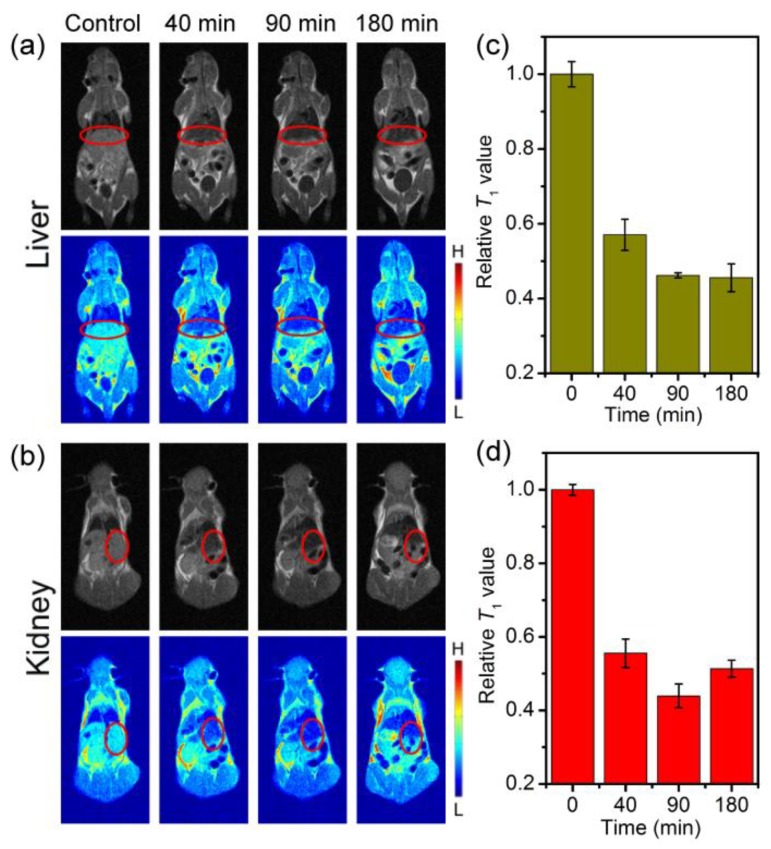
In vivo *T*_1_-weighted magnetic resonance (MR) images of mice (**a**) liver and (**b**) kidney (selected area) collected before (control group) and after intravenous injection of Fe_3_O_4_-BSA nanoparticles at different time points, and the corresponding relative *T*_1_-weighted signals extracted from (**c**) liver and (**d**) kidney sites.

To compare the MR contrast effect of Fe_3_O_4_-BSA and Fe_3_O_4_-PMAA-PTTM, in vivo *T*_1_-weight imaging experiment were performed on a 0.5 T MRI scanner using mice as model. After intravenous injection of Fe_3_O_4_-BSA/Fe_3_O_4_-PMAA-PTTM with a dose of 10 mg [Fe]/Kg [mouse], *T*_1_-weight MR images of the coronal planes of mice were acquired at different time points. For Fe_3_O_4_-BSA, darkening images were observed for the liver and kidney sites after intravenous injection for about 40 min (Figure 5a–b), indicating the Fe_3_O_4_-BSA displayed *T*_2_ contrast enhancement rather than *T*_1_ contrast enhancement in vivo. To quantify the contrast, the signal-to-noise ratio was calculated by analyzing the liver and kidney sites and the normal tissues of the MR image. As showed in Figure 5c–d, the relative *T*_1_ signals of liver and kidney decreased over time, and reached decreased maximum of at about 180 and 90 min with signals dropped of approximately 55% and 57% for liver and kidney, respectively. The decreased *T*_1_ signals can be can reasonably attributed to the slowly retention and aggregation of Fe_3_O_4_-BSA nanoparticles in liver and kidney sites, and consequently leading to the change of Fe_3_O_4_-BSA from *T*_1_ to *T*_2_ contrast agent. After intravenous injection of Fe_3_O_4_-BSA for about 180 min, the *T*_1_ signals of the kidney sites was slowly recovered to 51%, indicating the slowly metabolism of the Fe_3_O_4_-BSA from the mice.

**Figure 6:**
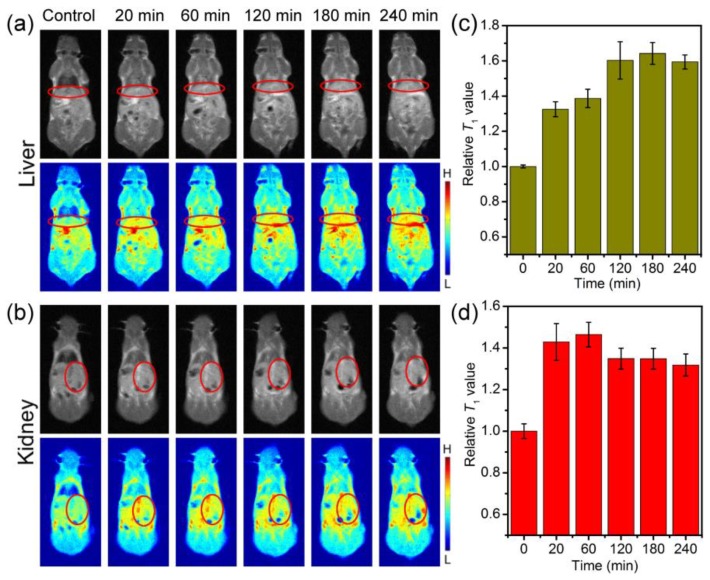
In vivo *T*_1_-weighted MR images of mice (**a**) liver and (**b**) kidney (selected area) collected before (control group) and after intravenous injection of Fe_3_O_4_-PMAA-PTTM nanoparticles at different time points, and the corresponding relative *T*_1_-weighted signals extracted from (**c**) liver and (**d**) kidney sites.

Different from Fe_3_O_4_-BSA, brighter images were observed at the liver and kidney sites after intravenous injection for 20 min, indicating that it can enhance *T*_1_ relaxation in vivo (Figure 6a–b). The relative signals extracted from the liver and kidney sites revealed that the signal enhancement in the liver increased over time and reached increased maximum of about 64% at 180 min, while that for the kidney achieved increased maximum of about 47% at 60 min, and then slowly decreased to about 32% at 240 min (Figure 6c–d). The contrast enhancements should be attributed to the slow retention, but not the aggregation, of Fe_3_O_4_-PMAA-PTTM nanoparticles in the liver and kidney sites. The *T*_1_ signals for liver sites decreased slowly from 240 min, respectively, which indicated the metabolism of the Fe_3_O_4_-PMAA-PTTM nanoparticles from the mice. These different MRI performances of Fe_3_O_4_-BSA and Fe_3_O_4_-PMAA-PTTM suggested that the ligands of the surface of nanoparticles play an important role in the optimizing the *T*_1_ MRI behaviors of nanoparticles in vivo.

## 4. Conclusions

In summary, we have used PMAA-PTTM and BSA as surface ligands to fabricate Fe_3_O_4_-BSA and Fe_3_O_4_-PMAA-PTTM nanoparticles with similar size and magnetization by the coprecipitation method and compared their MRI performances in vitro and in vivo. In vitro MRI experiments revealed that Fe_3_O_4_-PMAA-PTTM has lower *r*_2_/*r*_1_ ratios than that of Fe_3_O_4_-BSA, but both nanoparticles display *T*_1_-weight contrast enhancement in the solution (under experimental concentration). In vivo *T*_1_-weight imaging revealed that Fe_3_O_4_-BSA exhibits *T*_2_ contrast enhancement, while Fe_3_O_4_-PMAA-PTTM exhibits *T*_1_ contrast enhancement at liver and kidney sites, demonstrating that the surface ligands play an important role for the MRI performance of nanoparticles in vivo, which may shed some light on the design of macromolecule ligands for developing an iron oxide–based *T*_1_-weight contrast agent.
